# Synthesis of DHTA-Modified Poly(Epoxysuccinic Acid) and Scale Inhibition of Fluoride Scale

**DOI:** 10.3390/ma18204701

**Published:** 2025-10-14

**Authors:** Yihao Zhang, Bo Liu, Xiaolong Zhu, Chunxia Zhao, Zhe Qin, Zixue Liu, Da Lu

**Affiliations:** 1 Hebei Geo-Environment Monitoring, Shijiazhuang 050021, China; jcyzyh24@163.com (Y.Z.); hkyzxl@163.com (X.Z.); 2 Hebei Key Laboratory of Geological Resources and Environment Monitoring and Protection, Shijiazhuang 050021, China; 3 School of Eco-Environment, Hebei University, Baoding 071000, China; liubohd2025@163.com (B.L.); zhaoicx@126.com (C.Z.); qinzhe945@163.com (Z.Q.); wenyuan_4508@163.com (Z.L.)

**Keywords:** DHTA, polyepoxysuccinic acid, calcium fluoride, dynamic experiment, scale inhibition mechanism

## Abstract

To alleviate CaF_2_ scaling on reverse osmosis membranes, polyepoxysuccinic acid (PESA) was modified with 2,5-dihydroxyterephthalic acid (DHTA) to obtain DHTA-PESA. Its structure and thermal stability were confirmed through characterization. Scale inhibition performance was evaluated using static and dynamic experiments. Results showed that in static tests, at a dosage of 200 mg/L, DHTA-PESA achieved a CaF_2_ scale inhibition rate of nearly 100%, demonstrating Ca^2+^ chelation ability and the capacity to prolong crystallization induction time. In dynamic experiments, indicating superior CaF_2_ dispersion performance and effective mitigation of membrane fouling. X-ray diffraction and scanning electron microscopy analysis revealed that DHTA-PESA induces irregular growth of CaF_2_ crystals, disrupting their formation and altering crystal morphology. The primary scale inhibition mechanisms include dispersion, lattice distortion, and chelation.

## 1. Introduction

With the rapid development of modern industry, the problem of water scarcity has become increasingly severe. Water treatment technology, especially membrane treatment technology, has been recognized as an effective solution to this issue. Meanwhile, energy consumption and economic costs in the water treatment process can be significantly reduced by membrane treatment technology [1,2,3]. However, during the membrane treatment process, inorganic salt ions in water are concentrated and deposited as scale on the membrane surface. As a result, membrane pores are blocked. This phenomenon not only decreases treatment efficiency and increases energy consumption but also causes membrane pollution and shortens its service life. Adding scale inhibitors to the water circulation system has been regarded as one of the most economical and efficient approaches [4,5].

Among various scale inhibitors, polyepoxysuccinic acid (PESA) is widely applied in the field of scale inhibition. This is because its molecular structure contains polar carboxylic acid groups and functional groups such as hydroxyl groups [6]. Nevertheless, when PESA is used alone, its scale inhibition performance is often unsatisfactory. Therefore, PESA is frequently modified or compounded before applications [2,3].

Arg-PESA was synthesized through copolymerization of PESA and arginine (Arg). Evaluation results demonstrated that at 6 mg/L concentration, it achieved 100% scale inhibition efficiency against calcium carbonate, while reaching over 90% inhibition for calcium phosphate at 10 mg/L [7]. In a separate modification approach, amino acid (AA) was employed as an environmentally friendly grafting agent to functionalize PESA, yielding AA-PESA. This derivative effectively controls nucleation sites of CaCO_3_ crystals and significantly modulates the growth orientation of calcite (104) planes, ultimately attaining 100% scale inhibition performance against CaCO_3_ deposits [8]. SEA-PESA was synthesized using PESA and 2-aminoethanesulfonic acid (SEA) as monomers. When the mass concentration was 10 mg/L, the scale inhibition rate of calcium carbonate was close to 100% [9]. LAr PESA was prepared using raw materials such as maleic anhydride (MA) and L-arginine (LAr). When added at a concentration of 8 mg/L, the scale inhibition rates for CaCO_3_ and CaSO_4_ reached 91.2% and 94.5%, respectively [10]. By modifying PESA with thiourea (CNS), the scale inhibition rates of calcium carbonate and calcium phosphate were increased by 6% and 18.7%, respectively, compared to PESA, while exhibiting good corrosion inhibition performance [11]. The hyperbranched reaction of glycerol and PESA was carried out, and at a dosage of 15 mg/L, the scale inhibition rates of CaCO_3_ and CaSO_4_ reached 95.9% and 94.3%, respectively [12].

At present, more research has been conducted on scale inhibitors for CaCO_3_ and CaSO_4_, while relatively less research has focused on those for calcium fluoride (CaF_2_). With the continuous development of industrialization, circulating cooling water in steel plants contains large amounts of fluoride ions (F^−^). These ions form fluoride scale on reverse osmosis membrane equipment. This fluoride scale not only causes corrosion and deformation of some continuous casting components in pipelines but also leads to blockage of reverse osmosis membranes. As a result, desalination efficiency decreases and osmotic pressure increases, which affects the normal operation of the entire system. The service life of reverse osmosis membranes can also be shortened by fluoride scale. Due to the angular nature of CaF_2_ crystals, scratches are easily caused on the membrane surface during deposition. In addition, scaling leads to increased wear and corrosion on the membrane surface, resulting in irreversible damage to the equipment [13].

In this article, a modified polyepoxysuccinic acid scale inhibitor, DHTA-PESA, was synthesized to address the membrane fouling problem caused by CaF_2_ scaling. The structure and properties of DHTA-PESA were explored.

## 2. Materials and Methods

### 2.1. Main Reagents and Instruments

All chemicals were used as received without further purification. Maleic anhydride (C_4_H_2_O_3_), sodium hydroxide (NaOH), calcium hydroxide (Ca(OH)_2_), hydrogen peroxide (H_2_O_2_, 30%), sodium tungstate (Na_2_WO_4_), trisodium citrate dihydrate (Na_3_C_6_H_5_O_7_·2H_2_O), anhydrous calcium chloride (CaCl_2_), sodium tetraborate (Na_2_B_4_O_7_), glacial acetic acid (C_2_H_4_O_2_), and sodium fluoride (NaF) were purchased from Tianjin Kemiou Chemical Reagent Co., Ltd. (Tianjin, China), sodium hydroxide (NaOH), calcium hydroxide (Ca(OH)_2_), and sodium tungstate (Na_2_WO_4_) were also purchased from Tianjin Damao Chemical Reagent Factory. 2,5-Dihydroxyterephthalic acid (C_8_H_6_O_6_) was purchased from Shanghai Macklin Biochemical Co., Ltd. (Shanghai, China). Methanol (CH_4_O) was purchased from Tianjin Zonghengxing Industry & Trade Co., Ltd. (Tianjin, China). Sodium chloride (NaCl) was purchased from Tianjin Huihang Chemical Technology Co., Ltd. (Tianjin, China). All compounds were of analytical reagent (AR) grade.

**Figure 1:**
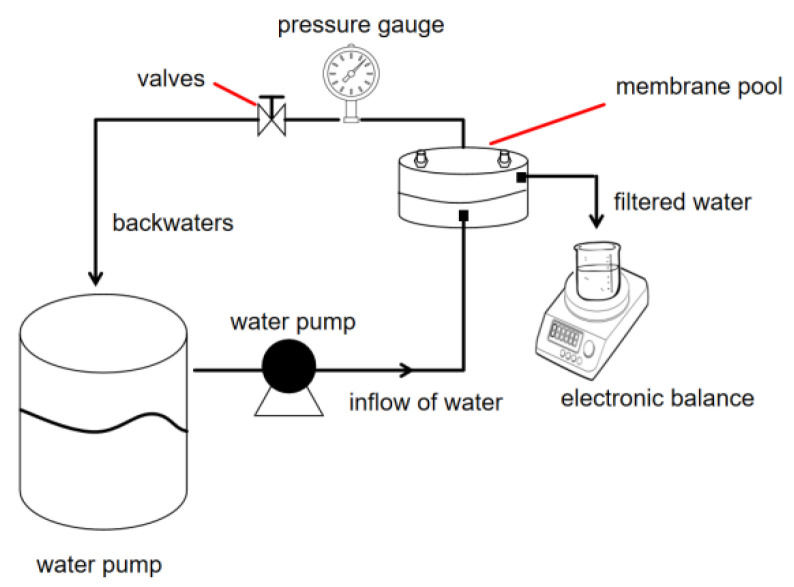
Dynamic experimental setup diagram.

The major instruments employed were as follows: Fourier-transform infrared spectrometer (FT-IR, Nicolet Is 10, Thermo Fisher Scientific, Waltham, MA, USA; wavenumber range: 600–4000 cm^−1^), nuclear magnetic resonance spectrometer (^1^H NMR, AVANCE III 500, Bruker, Berlin, Germany; solvent: D_2_O), Thermogravimetric analyzer (TGA, STA 449C, Netzsch, Selb, Germany; temperature range: 35–400 °C, heating rate: 20 °C/min), scanning electron microscope (SEM, Phenom-World, Eindhoven, The Netherlands; magnification: 500×), X-ray diffractometer (XRD, DX-2700, Dandong Haoyuan Instrument Co., Ltd., Dandong, China; scan rate: 15°/min, 2θ range: 5–85°), X-ray photoelectron spectrometer (XPS, Thermo Fisher Scientific, Waltham, MA, USA), fluoride ion selective electrode (used with a suitable meter, details needed*), conductivity meter (HQ30d, Hach, Loveland, LO, USA), and a custom-built dynamic scale inhibition test setup (as depicted in Figure 1).

### 2.2. Synthesis of Scale Inhibitor

For this, 24.5 g of maleic anhydride was added to a three-necked flask and dissolved in 10 mL of water. Then, 30 mL of 50% NaOH solution was introduced, and the mixture was heated to 55 °C. Subsequently, 2 g of sodium tungstate and 25 mL of 30% H_2_O_2_ were added. The pH of the solution was adjusted to 6, and the temperature was raised to 65 °C for a 1.5 h reaction, yielding cyclo[oxysuccinic acid] (ESA). The temperature was then increased to 90 °C, and the pH was adjusted to 12. Calcium hydroxide (Ca(OH)_2_) was added, followed by a 3 h reaction to obtain polyepoxysuccinic acid (PESA).

**Figure 2:**
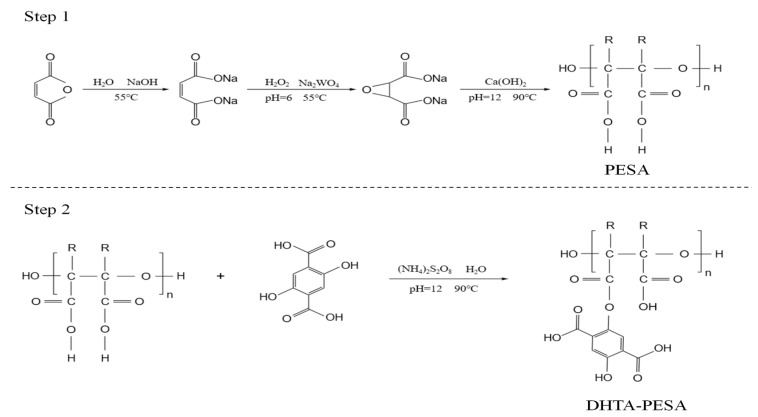
Reaction process during the preparation of DHTA-PESA.

Here, 6 g of the as-prepared PESA was dissolved in water in a three-necked flask at 45 °C. 2,5-Dihydroxyterephthalic acid (DHTA) and 0.03 g of initiator ammonium persulfate were added. The pH was adjusted to 12, and the temperature was raised to 90 °C for a 3 h reaction, resulting in the preparation of the modified scale inhibitor DHTA-PESA containing epoxysuccinic acid groups. Figure 2 is the synthesis diagram of DHTA-PESA.

### 2.3. Characterization of Scale Inhibitors and Scale Samples

The synthesized scale inhibitor’s structure and thermal stability were analyzed. The Nicolet Is 10 infrared spectrometer from Thermo Fisher Scientific was used, with a wavenumber scanning range of 600–4000 cm^−1^. The AVANCE III 500 nuclear magnetic hydrogen spectrometer from Bruker, Berlin, Germany, was employed, using D_2_O as the solution. The STA 449C thermogravimetric analyzer from NETZSCH, Selb, Germany, was utilized, with a temperature range of 35–400 °C and a heating rate of 20 °C/min.

The CaF_2_ scale formed in static experiments was measured to explore the scale inhibition mechanism. A scanning electron microscope (magnification: 500×) from Phenom World (Eindhoven, The Netherlands) was used. An X-ray diffractometer from Dandong Haoyuan (scanning rate: 15 °/min within 5–85°) was employed. An X-ray photoelectron spectrometer from Thermo Fisher Scientific was utilized.

### 2.4. Viscosity-Average Molecular Weight Measurement

The molecular weights of scale inhibitors were determined using the viscosity method. Solutions of various scale inhibitors at 10 g/L were prepared with distilled water as the solvent. The time taken for distilled water to pass through the timing line was measured using a stopwatch, and the average of three measurements was recorded as *t*_0_. The viscosity-average relative molecular weight M was calculated using the Mark–Houwink equation.

### 2.5. Scale Inhibition Performance Evaluation

#### 2.5.1. Static Scale Inhibition Experiment

Twenty milliliters of 0.15 mol/L calcium chloride, the scale inhibitor, 20 mL of borax buffer solution, and 20 mL of 0.3 mol/L sodium fluoride were added to a 500 mL volumetric flask. The mixture was diluted to the mark with water and shaken well. The solution was heated in a constant-temperature water bath at 80 °C for 8 h and then removed. After standing at room temperature, the solution was filtered. Ten milliliters of the supernatant and 10 mL of TISAB (fluoride ion buffer solution) were taken and diluted to 50 mL, followed by shaking to mix uniformly. The fluoride ion content was measured using an electrode, and the scale inhibition rate was calculated with the following formula:

(1) $$lgC_{F^{-}}=\frac{\left(E-50.8\right)}{-55.2}$$

(2) $$\beta =\frac{\left(0.012-5*F_{\;blank}^{-}\right)-\left(0.012-F_{measurement}^{-}\right)}{0.012-5*F_{\;blank}^{-}}$$

where

$C_{F^{-}}$: the concentration of fluoride ions in the solution, mol/L.

E: measured electromotive force, mv.

0.012: total fluoride ion concentration added, mol/L.

$F_{\;blank}^{-}$: fluoride ion concentration measured in the blank, mol/L.

$F_{measurement}^{-}$: the fluoride ion concentration measured by the experimental group, mol/L.

β: scale inhibition rate.

50.8, 55.2: from the fitting equation.

#### 2.5.2. Conductivity Measurement

The scale inhibitor was mixed into the sodium fluoride solution. Calcium chloride was added to achieve a Ca^2+^ concentration of 240 mg/L. The pH was adjusted to 8.0, and the solution temperature was maintained at 25 °C. The conductivity of the solution was measured using an HQ30d conductivity meter (Hach Corporation, Loveland, CO, USA). The time point at which the conductivity significantly decreased was defined as the crystallization induction time.

#### 2.5.3. Dynamic Experiment


(3) $$J_{t}/J_{0}=\frac{m_{t}/∆t}{m_{0}/t_{0}}$$

As shown in Figure 1, the water tank had a volume of 500 mL, a Ca^2+^ concentration of 0.4 mol/L, and an F^−^ concentration of 0.8 mol/L. The membrane cell had an effective diameter of 60 mm, a permeation pressure of 0.7 MPa, and a temperature of approximately 25 °C. The mass change in filtered water per minute was read using an electronic balance to calculate the membrane-specific flux.

The membrane-specific flux is a parameter for measuring membrane working conditions and evaluating the scale inhibition performance of scale inhibitors. The membrane-specific flux (*J_t_*/*J*_0_) was calculated using Equation (3).

*J_t_*: permeate flux at time *t*, (m^3^/(min·m^2^)).

*m_t_*: permeate mass during Δ*t* at time *t*, (g).

*m*_0_: initial permeate mass during Δ*t*_0_, (g).

## 3. Results and Discussion

### 3.1. Optimization of Synthesis Conditions

**Figure 3:**
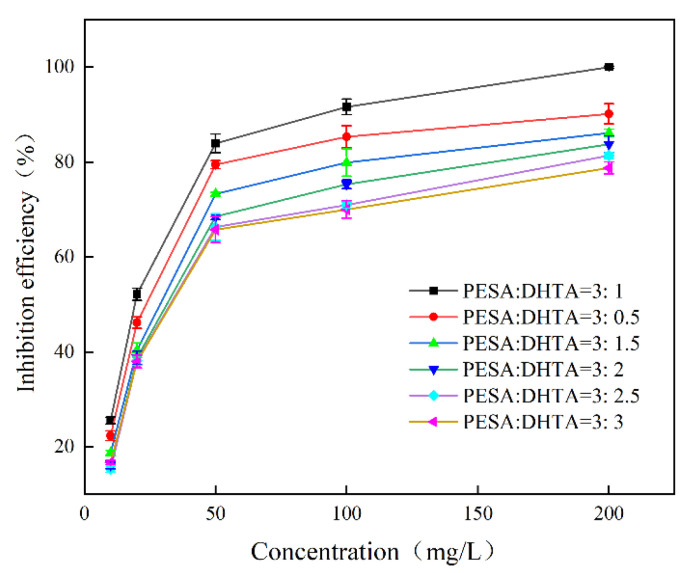
Effect of different raw material ratios on scale inhibition rate.

To explore the optimal DHTA:PESA ratio for the CaF_2_ scale inhibitor, six groups of scale inhibitors with PESA ratios of 3:0.5, 3:1, 3:1.5, 3:2, 3:2.5, and 3:3 were prepared. The variance plot of the effect of scale inhibition performance is shown in Figure 3.

With the increase in the concentration of scale inhibitors, all scale inhibition rates exhibit an upward trend. The introduced carboxyl and hydroxyl groups are strongly chelated with Ca^2+^, directly and effectively inhibiting the formation of CaF_2_ crystals [14].

When the mass ratio of PESA to DHTA is set as 3:1, the highest scale inhibition rate is exhibited by DHTA-PESA. A high PESA-to-DHTA ratio is accompanied by shorter grafting chains of PESA, and a weaker chelating effect is caused accordingly. As the dosage of DHTA is increased, the graft chains are allowed to grow continuously, and the appropriate amounts of carboxylic acid groups and hydroxyl groups are increased, which is beneficial to chelation and contributes to the improvement in scale inhibition performance. When the grafting chains become excessively long, the molecular weight is increased, and intermolecular hydrogen bonds are prone to be formed by DHTA-PESA molecules, which is unfavorable for chelation with Ca^2+^. In addition, longer polymer chains can promote flocculation and bridging between particles, and the formation of scale is led to [15]. Therefore, the optimal scale inhibition performance is demonstrated by the PESA-to-DHTA mass ratio of 3:1.

### 3.2. Performance Characterization of Synthesized Products

#### 3.2.1. Infrared Spectroscopy

**Figure 4:**
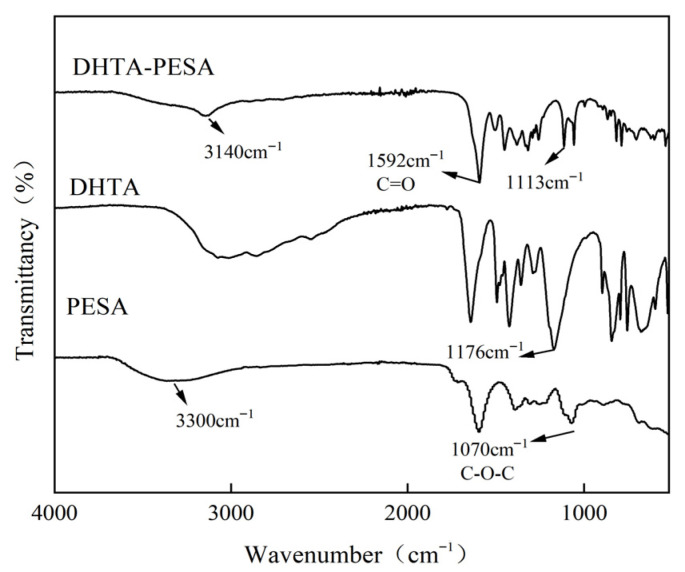
Infrared spectra of PESA, DHTA, and DHTA-PESA.

Infrared spectroscopy measurement is carried out on a scale inhibitor with a PESA:DHTA ratio of 3:1, as shown in Figure 4.

The stretching vibration absorption peak of the hydroxyl group (O-H) in the carboxyl (–COOH) functional group of PESA is observed at 3300 cm^−1^. For the modified DHTA-PESA, a hydroxyl stretching vibration peak is also detected at 3140 cm^−1^ [16], and a distinct characteristic peak is identified at 1592 cm^−1^, which is assigned to the carbonyl(C=O) stretching vibration absorption peak of the introduced carboxylic acid group. The presence of carboxyl and hydroxyl groups in the product is confirmed [17]. By comparison with the characteristic peaks of PESA, the characteristic peaks of DHTA-PESA are found to be more significant. It is thus demonstrated that carboxyl and hydroxyl groups have been introduced and increased.

#### 3.2.2. Nuclear Magnetic Hydrogen Spectroscopy

**Figure 5:**
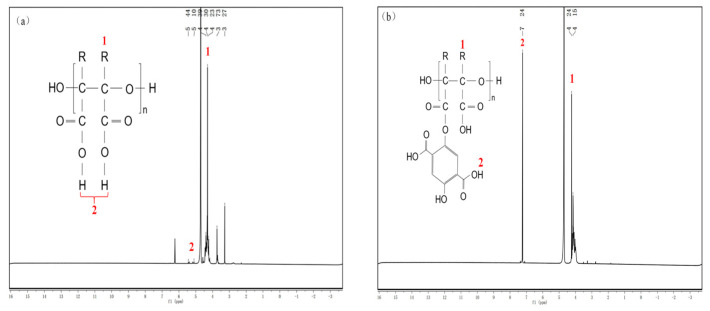
NMR hydrogen spectra of the products: (**a**) NMH spectrum of PESA and (**b**) NMH spectrum of DHTA-PESA.

To verify the correctness of the modified product synthesis, ^1^HNMR testing was conducted. The results are shown in Figure 5.

In Figure 5a, the hydrogen peak of the secondary chain-end CH is observed at a chemical shift of δ = 4.30, and the hydrogen peak of the chain-end O-H is detected at chemical shifts of δ = 5.10–5.46, indicating that PESA was successfully synthesized.

In addition to the characteristic peaks contained in PESA, the modified product in Figure 5b also has a peak of H in the carboxyl group on the benzene ring of δ = 7.24 ppm. The reaction between the hydroxyl group on the DHTA benzene ring and the carboxyl group on the PESA side chain leads to the presence of the benzene ring in the structure of the modified product.

Combined with the results of infrared spectroscopy, it is demonstrated that DHTA-PESA possesses the functional groups of the target product.

#### 3.2.3. Molecular Weight Determination

The time for DHTA-PESA to flow through the marked lines is substituted into Equations (1)–(3), and the relevant viscosity data are calculated. The results are shown in Table 1 and Table 2.

The average of three relative molecular weights was calculated, and MPESA = 478.69 and MDHTA-PESA = 1548.50 were obtained. The molecular weight of PESA was increased by the introduction of DHTA. The successful synthesis of DHTA-PESA was verified by infrared spectroscopy and nuclear magnetic hydrogen spectroscopy.

#### 3.2.4. Thermal Stability Analysis

**Figure 6:**
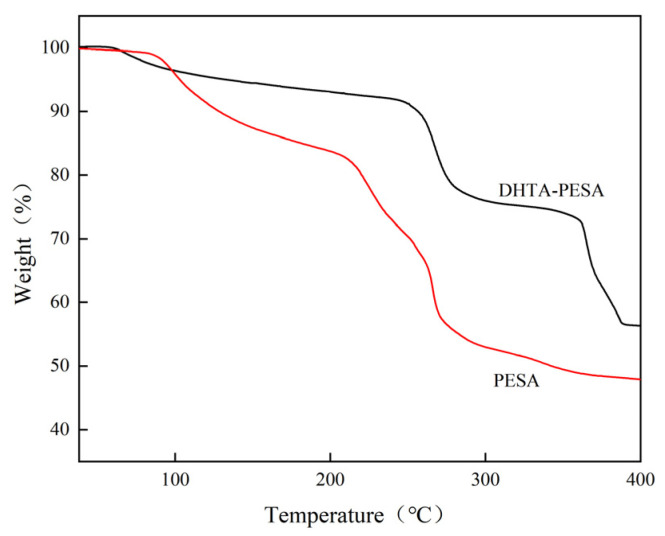
Thermogravimetric analysis curves of PESA and DHTA-PESA.

Compare the thermogravimetric curve of DHTA-PESA with PESA to determine its thermal stability, as shown in Figure 6.

As shown in Figure 6, approximately 15% weight loss of PESA is observed in the temperature range of 35–200 °C, which is mainly attributed to the evaporation of intermolecular water and the decomposition of oligomers. Within the temperature range of 200–280 °C chemical bonds of PESA are broken [18]. Finally, the mass of the polymer decreases slowly between 280 and 400 °C and enters a stable stage. The mass loss process of DHTA-PESA is also divided into two parts, with minimal loss observed before 250 °C, indicating excellent thermal stability. Internal chemical bond breakage occurs only around 350 °C, and the final mass can still be maintained at approximately 60%.

Good thermal stability and a more stable structure are exhibited by DHTA-PESA.

### 3.3. Scale Inhibition Performance

#### 3.3.1. Static Scale Inhibition

(1) The influence of concentration on scale inhibition rate

**Figure 7:**
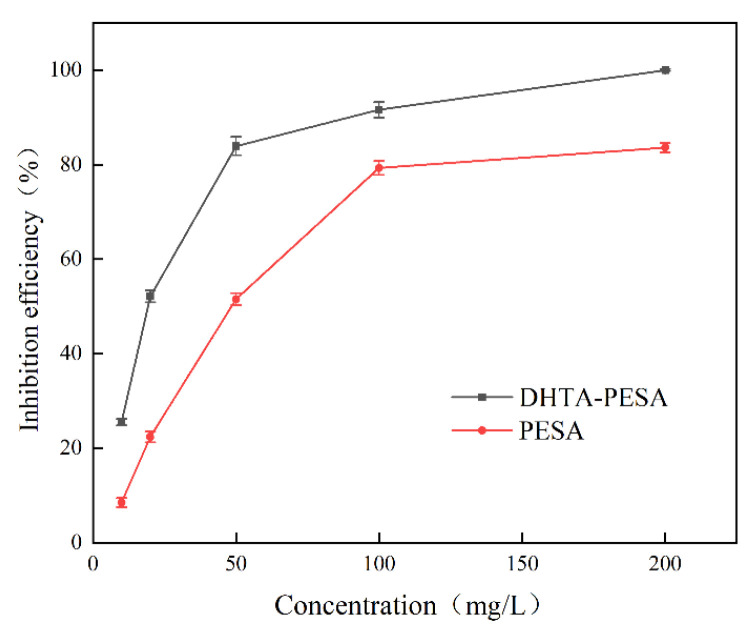
Effect of concentrations on scale inhibition rates.

Static scale inhibition experiments were performed to evaluate the efficacy of DHTA-PESA at various concentrations in inhibiting CaF_2_ scale formation, with PESA used as a control. The results are presented in Figure 7. The simulated water quality conditions were as follows: C(Ca^2+^) = 240 mg/L, pH = 7, and a constant temperature of 80 °C for 8 h.

The impact of varying DHTA-PESA concentrations on the scale inhibition efficiency of CaF_2_ was investigated. It was found that the scale inhibition rate of DHTA-PESA increases with increasing concentration. As the concentration rises, the number of functional groups increases, leading to enhanced chelation of Ca^2+^ ions, which in turn inhibits the formation of CaF_2_ scale. Simultaneously, carboxyl groups can effectively adsorb onto the surface of CaF_2_ crystals and, through charge repulsion, exert favorable repulsive, dispersive, and compatibilizing effects on the generated CaF_2_ microcrystals. The dispersion effect becomes more pronounced at higher concentrations [19,20].

When the concentration exceeds 50 mg/L, the growth rate of the scale inhibition rate is slowed down. This is due to the existence of a “threshold effect”, where the Ca^2+^ content in water and the number of active sites on the surface of CaF_2_ crystals are limited. When the concentration reaches a certain level, increasing the concentration of the scale inhibitor cannot change the number of active sites and thus cannot enhance the scale inhibition performance, resulting in a threshold [21]. The scale inhibition rate of DHTA-PESA is consistently higher than that of PESA, indicating that the introduction of a large number of carboxyl groups contributes to the inhibition of CaF_2_ scaling.

Considering cost-effectiveness and actual usage [22,23], 100 mg/L was selected as the concentration for subsequent research.

(2) Influence of Temperature on Scale Inhibition Rate

**Figure 8:**
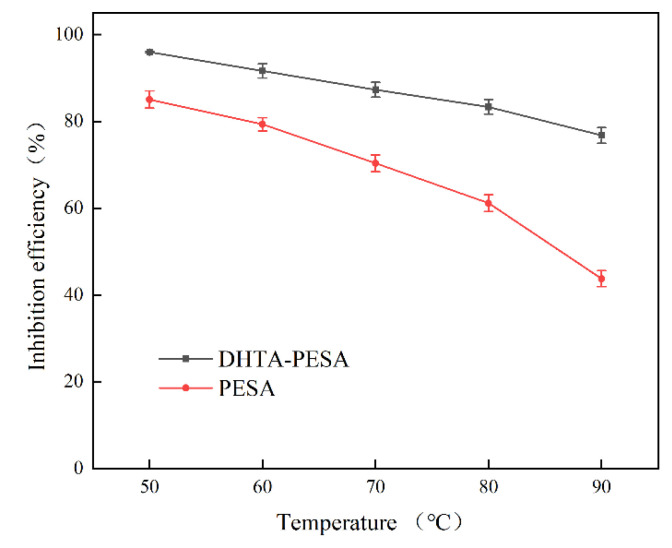
Effect of temperature on scale inhibition rate.

To further investigate the scale inhibition effect and heat resistance of DHTA-PESA, the effects of different temperatures on its scale inhibition performance were examined. The experiments were conducted at a scale inhibitor dosage of 100 mg/L, a solution concentration of C (Ca^2+^) = 240 mg/L, a constant temperature duration of 8 h, and a pH of 7. The results are presented in Figure 8.

The scale inhibition rate of DHTA-PESA decreases continuously as temperature increases. This is because the intensification of temperature leads to enhanced movement of various ions in water, increasing the collision probability between Ca^2+^ and F^−^. This promotes their combination to form CaF_2_ precipitation, thereby reducing the scale inhibition rate. Meanwhile, with the increase in reaction temperature, DHTA-PESA undergoes decomposition, and the main chain of the polymer may break [24]. This results in the destruction of scale inhibition functional groups and a decline in scale inhibition performance. Additionally, decomposed substances generate interference in the solution, inhibiting the scale inhibition process and decreasing the scale inhibition efficiency of DHTA-PESA.

At 50 °C, DHTA-PESA not only exhibits a high-scale inhibition rate but also is closer to the actual conditions of circulating water. Therefore, 50 °C was selected as the heating temperature for subsequent research.

(3) Influence of Calcium Ion Concentration on Scale Inhibition Rate

**Figure 9:**
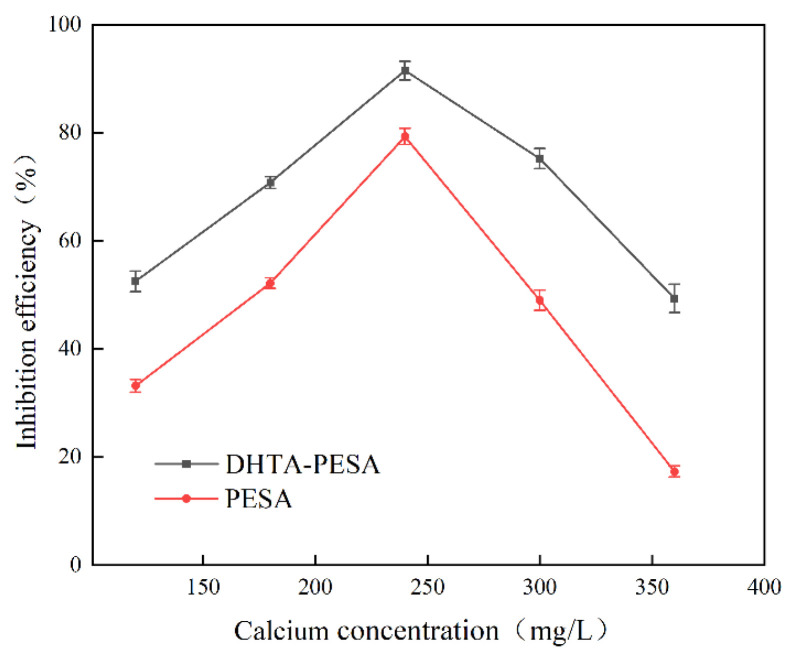
Effect of Ca^2+^ concentration on scale inhibition rate.

The effects of different Ca^2+^ concentrations on the scale inhibition performance of DHTA-PESA were investigated under the conditions of a scale inhibitor concentration of 100 mg/L, pH = 7, and a constant temperature of 50 °C for 8 h. The experimental results are shown in Figure 9.

Figure 9 depicts the impact of Ca^2+^ concentration on scale inhibition efficiency. A trend of the scale inhibition rate first increasing and then decreasing was observed. The peak scale inhibition rate of DHTA-PESA was achieved at a Ca^2+^ concentration of 240 mg/L. This phenomenon is attributed to the chelating effect of the scale inhibitor, which can bind most Ca^2+^ ions at lower concentrations. As the concentration rises, the chelating effect is enhanced, leading to an increased scale inhibition rate. However, the chelating capacity of the scale inhibitor is limited. When the Ca^2+^ concentration exceeds this limit, the excess Ca^2+^ ions cannot be bound, resulting in a decline in the scale inhibition rate [25]. DHTA-PESA exhibits superior scale inhibition ability compared to PESA, suggesting that the modified carboxyl and hydroxyl groups can chelate more Ca^2+^ ions and exert a stronger inhibitory effect on CaF_2_ formation.

Given that DHTA-PESA demonstrates the optimal scale inhibition rate at a calcium ion concentration of 240 mg/L, this concentration was selected as a water quality condition for further research.

(4) Effect of Heating Time on Scale Inhibition Rate

**Figure 10:**
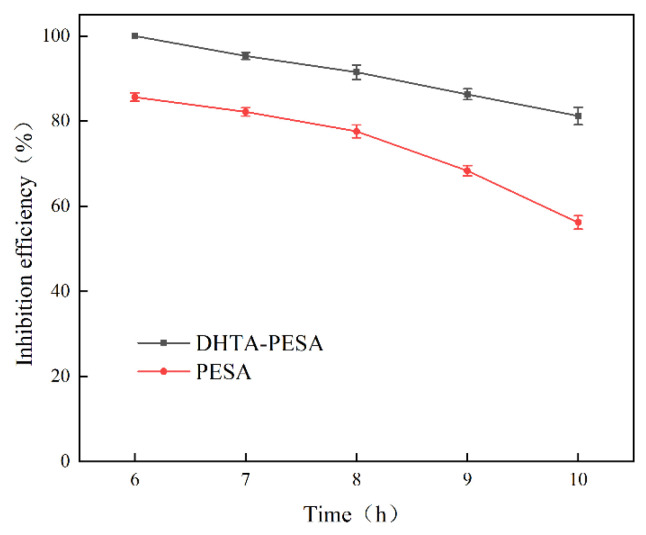
Effect of scale inhibition time on scale inhibition rate.

To investigate the persistence of DHTA-PESA’s scale inhibition performance against CaF_2_, the effects of different heating times were studied. Experiments were conducted at a scale inhibitor concentration of 100 mg/L, water quality conditions of pH = 7, Ca^2+^ concentration of 240 mg/L, and a heating temperature of 50 °C. The results are shown in Figure 10.

As shown in Figure 10, the scale inhibition rate of DHTA-PESA approaches 100% after heating at 50 °C for 6 h. However, the scale inhibition rate gradually decreases as the heating time is extended. This phenomenon is attributed to the gradual increase in the ability of the scale inhibitor to adsorb and disperse Ca^2+^ ions before reaching a certain constant temperature time, leading to an enhanced scale inhibition rate. When the time exceeds 6 h, the amount of calcium ions that can be bound in the solution gradually decreases, causing the equilibrium of the complexation reaction to shift in the reverse direction and resulting in a continuous decline in the scale inhibition rate [26]. The scale inhibition rate of PESA decreased by approximately 40%, indicating poor persistence. This is because PESA contains a limited number of functional groups, which are continuously consumed over time, leading to a decreasing scale inhibition rate. In contrast, DHTA-PESA exhibits a high functional group content, enabling it to effectively inhibit scaling over an extended period.

Based on the actual water usage time and experimental results, 8 h was selected as the heating time for subsequent research.

(5) Influence of pH on Scale Inhibition Rate

**Figure 11:**
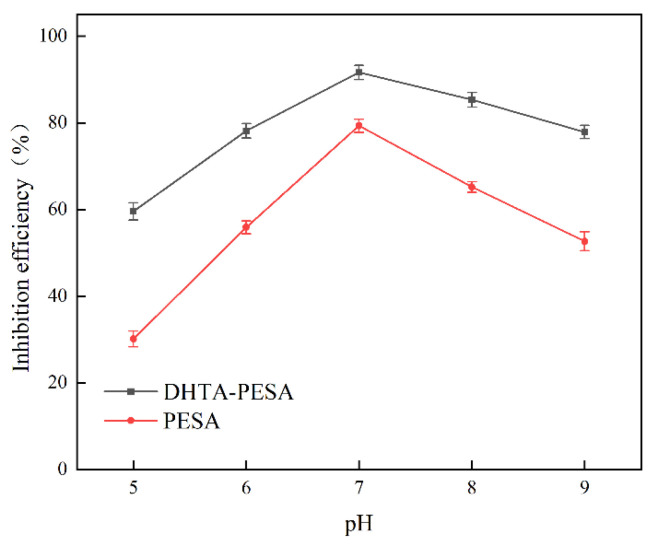
Effect of pH on scale inhibition rate.

To investigate the alkali resistance of DHTA-PESA, the effects of pH ranging from 5 to 9 on its scale inhibition performance were examined. Experiments were conducted under the conditions of a scale inhibitor concentration of 100 mg/L, a Ca^2+^ concentration of 240 mg/L, and a constant temperature of 50 °C for 8 h. The results are shown in Figure 11.

Figure 11 illustrates the effect of pH on the scale inhibitor performance. As the pH value increases, the scale inhibition rate first increases and then decreases, with the optimal rate achieved at pH = 7. Overall, the scale inhibitors demonstrate better scale inhibition performance at higher pH values, which is attributed to the stronger ionization of carboxyl groups on the scale inhibitors and the higher electrostatic repulsion ability at higher pH.

Based on the above analysis and actual conditions, DHTA-PESA exhibits the optimal scale inhibition rate under the following conditions: a concentration of 100 mg/L, a temperature of 50 °C, a calcium ion (Ca^2+^) concentration of 240 mg/L, a heating time of 6 h, and pH = 7.

(6) Static conductance experiment

**Figure 12:**
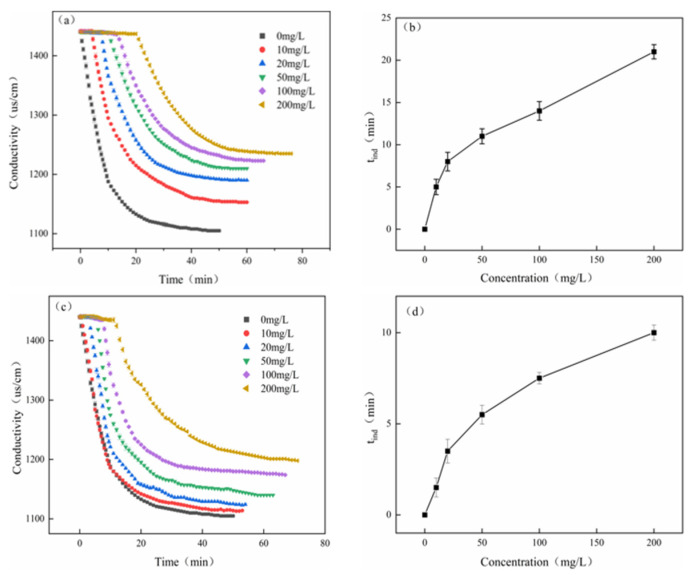
Conductivity change in the solution and the corresponding t_ind_ change after adding different concentrations of scale inhibitors. (**a**,**c**) Changes in conductivity of solutions after dosing different concentrations of PESA and DHTA-PESA and (**b**,**d**) corresponding change in t_ind._

In this study, a solution with a Ca^2+^ concentration of 240 mg/L was selected, and different concentrations of DHTA-PESA were added to it. The effect of DHTA-PESA concentration on the crystallization process of calcium fluoride was investigated via conductivity and induction time measurements. The results are shown in Figure 12.

Figure 12a,c illustrate the effect of concentration on conductivity. The conductivity of DHTA-PESA increases with increasing concentration. This is because as the concentration increases, the number of functional groups increases [27], more F exists in water in a free state, the ion content in water increases, and the conductivity increases. However, the conductivity of DHTA-PESA was significantly higher than that of PESA. The ability of DHTA-PESA to chelate Ca^2+^ was stronger, and the ion content in water was more, which made the conductivity increase significantly.

Figure 12b,d show the effect of scale inhibitor concentration on induction time. The induction time of DHTA-PESA is 21 min, compared to 12 min for PESA. DHTA-PESA significantly prolongs the induction time, and the increase in induction time indicates a slower crystal growth rate, which is crucial for delaying scaling in reverse osmosis systems. The excellent scale inhibition effect of DHTA-PESA is due to the carboxyl functional groups it contains, which not only enhance solubility but also exhibit a dispersing effect and prolong the induction time [28,29,30].

#### 3.3.2. Dynamic Scale Inhibition

**Figure 13:**
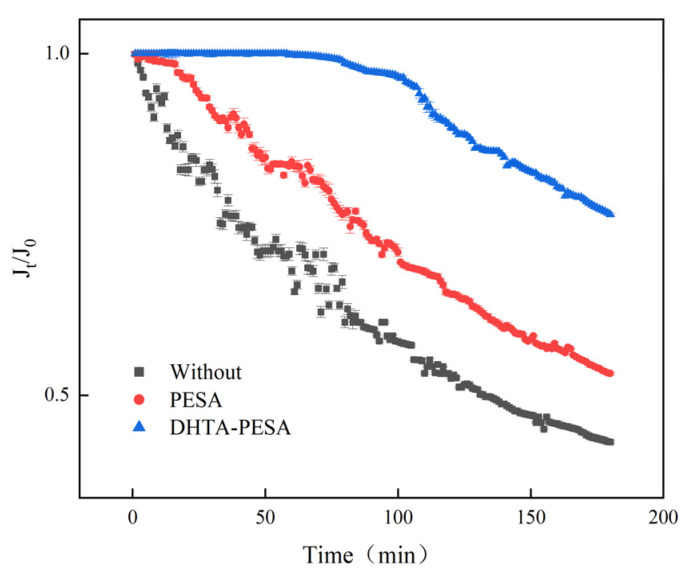
Membrane-specific flux curve over time.

The dynamic experiment realistically simulated the operational state of the reverse osmosis membrane in the circulating water system and evaluated the scale inhibition performance of DHTA-PESA in flowing water. Dynamic experiments with DHTA-PESA were conducted at a dosage of 100 mg/L and a runtime of 3 h. The specific membrane flux was calculated based on changes in effluent quality per unit time to assess the scale inhibition effect. The experimental results are shown in Figure 13. 

As shown in the figure, when no scale inhibitor is added, the specific membrane flux in the system decreases significantly and rapidly. This indicates that a large amount of CaF_2_ scale is quickly generated in the system, causing blockage of the reverse osmosis membrane. Additionally, the specific membrane flux in the system is unstable, exhibiting fluctuations between high and low values. Without a scale inhibitor, Ca^2+^ and F^−^ easily collide in water to form CaF_2_ scale, which blocks the membrane pool as it flows through, leading to a decrease in specific membrane flux. After CaF_2_ scale formation, the concentrations of Ca^2+^ and F^−^ in the system decrease, causing a short-term increase in specific membrane flux when no scale particles or fine particles flow through the membrane pool [31,32]. After the addition of DHTA-PESA, a dispersing effect is produced, resulting in a uniform ion concentration throughout the system and a reduction in CaF_2_ scale formation. This is manifested as a uniform and gradual decrease in specific membrane flux, demonstrating that DHTA-PESA inhibits CaF_2_ scale formation.

Compared with PESA, DHTA-PESA exhibits a lower and slower decline rate in specific membrane flux. The specific membrane flux without scale inhibitor decreases by 57%, while that with DHTA-PESA decreases by only 24% and shows a downward trend after 70 min. This indicates that DHTA-PESA has a good dispersing effect, leading to uniform concentration in the system, stable effluent, and effective inhibition and slowing of CaF_2_ scale formation. Due to the presence of a large number of carboxylic acid groups, DHTA-PESA can chelate with Ca^2+^ to form soluble polymers, which do not cause blockage to the reverse osmosis membrane within a certain period. However, with the passage of time, the functional groups of DHTA-PESA are exhausted and can no longer bind Ca^2+^. The remaining small amount of Ca^2+^ collides with F^−^ under the action of water flow to form CaF_2_ scale, resulting in a decrease in specific membrane flux after 70 min.

**Figure 14:**
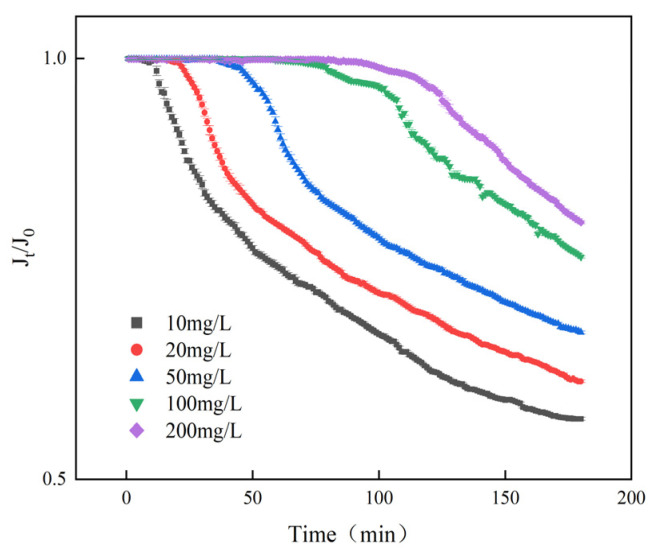
Effect of DHTA-PESA concentration on membrane-specific fluxes.

To further investigate the scale inhibition performance of DHTA-PESA, the effect of dosage on specific membrane flux was studied. The experimental results are shown in Figure 14.

As shown in the figure, the specific membrane flux increases with increasing concentration. A 23% increase in the specific membrane flux of DHTA-PESA is observed when the concentration is raised from 10 mg/L to 200 mg/L. This is because higher concentrations introduce more functional groups, enhancing chelating and dispersing effects. The carboxyl and hydroxyl groups in DHTA-PESA can chelate more Ca^2+^, inhibiting calcium fluoride scale formation and reducing membrane clogging, thereby leading to a slower decrease in specific membrane flux.

#### 3.3.3. Water Trial Experiment

**Figure 15:**
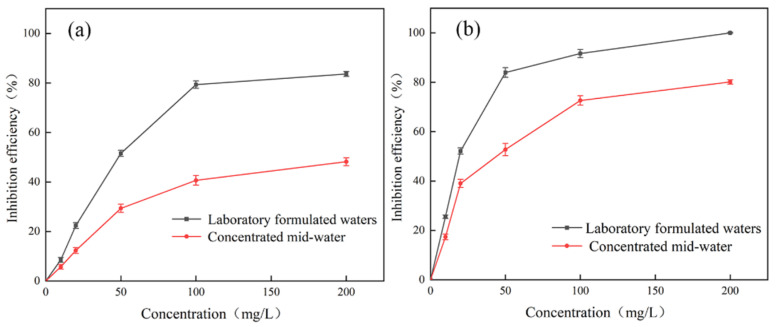
Scale inhibition performance test of PESA and DHTA-PESA in concentrated medium water: (**a**) PESA and (**b**) DHTA-PESA.

Static experiments were conducted using reverse osmosis water from steel mills. The water quality of the reclaimed water is shown in Table 3, and the results are shown in Figure 15.

As shown in the figure, the scale inhibition performance of PESA in concentrated water is significantly reduced. When the PESA concentration is 200 mg/L, the scale inhibition rate is approximately 50%. This is because the Ca^2+^ concentration in the concentrated water increases significantly, exceeding the chelation capacity of PESA, so excess Ca^2+^ cannot be bound, leading to a decrease in the scale inhibition rate. Meanwhile, the concentrated water contains a large number of interfering ions (such as Cl^−^, K^+^, Na^+^, etc.), which interfere with the chelation process and cause the scale inhibition rate to decline. In addition, the pH value of the actual water body is higher than that of the prepared water body, which affects the ionization and electrostatic repulsion ability of the scale inhibitor, reduces the amount of chelated Ca^2+^, and weakens the repulsion between CaF_2_ microcrystals, making CaF_2_ more prone to aggregation.

PESA also reaches the threshold earlier in the middle water. Within the concentration range of 50 mg/L to 200 mg/L, the scale inhibition rate only increases by 19%. Due to the limited number of active sites on the surface of microcrystals and the complex ion composition in the water, the formed CaF_2_ crystals are impure, leading to a reduction in the number of active sites on the microcrystals. As a result, the number of PESA molecules that can bind to the microcrystals decreases, causing the threshold to be reached earlier.

The decrease in the scale inhibition rate of DHTA-PESA is relatively small, and at a dosage of 200 mg/L, the scale inhibition rate can reach 80.15%. DHTA-PESA contains a large number of functional groups, which can bind more Ca^2+^ to reduce scale formation. This also indicates that the introduction of carboxylic acid groups helps to reduce the impact of water quality factors and enhance the inhibition capacity against CaF_2_ scale. Therefore, DHTA-PESA can adapt to more complex water bodies.

### 3.4. Analysis of Scale Inhibition Mechanism

#### 3.4.1. SEM Analysis

**Figure 16:**
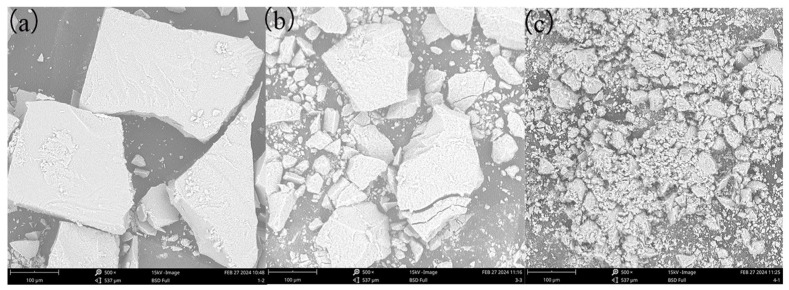
SEM images of CaF_2_ scale under different conditions (concentration is 100 mg/L): (**a**) CaF_2_ scale without scale inhibitor added; (**b**) CaF_2_ scale after addition of PESA; (**c**) CaF_2_ scale after addition of DHTA-PESA.

CaF_2_ scale before and after adding scale inhibitor was collected and dried, and SEM analysis was performed on it. The results are shown in Figure 16.

Without the addition of scale inhibitors, CaF_2_ crystals exhibit a dense cubic structure with clear boundaries and a relatively regular shape. As shown in Figure 16a, the crystal surface after adding PESA displays obvious loose pores, indicating lattice distortion. After adding DHTA-PESA, as shown in Figure 16b, CaF_2_ crystals transform into small particles with gradually blurred boundaries and rounded edges, becoming looser and less prone to aggregation. DHTA-PESA influences crystal formation by occupying active sites, causing crystals to grow in irregular directions. This ultimately results in the formation of loose and rounded small particles, which are more easily washed away by water flow and reduce scale formation.

SEM analysis reveals that DHTA-PESA exhibits a more pronounced scale inhibition effect. The introduction of carboxyl and hydroxyl groups enhances chelation with Ca^2+^ [33,34,35], forming water-soluble chelates that stabilize Ca^2+^ in solution and convert it into soluble salts, thereby reducing scale formation [36]. Carboxyl groups can also bind to CaF_2_ microcrystals, and the repulsive forces generated between adjacent carboxyl groups prevent microcrystal clustering and the formation of larger scales, achieving scale inhibition. The incorporation of carboxyl and hydroxyl groups is the primary factor contributing to the enhanced scale inhibition performance of DHTA-PESA.

#### 3.4.2. XRD Analysis

**Figure 17:**
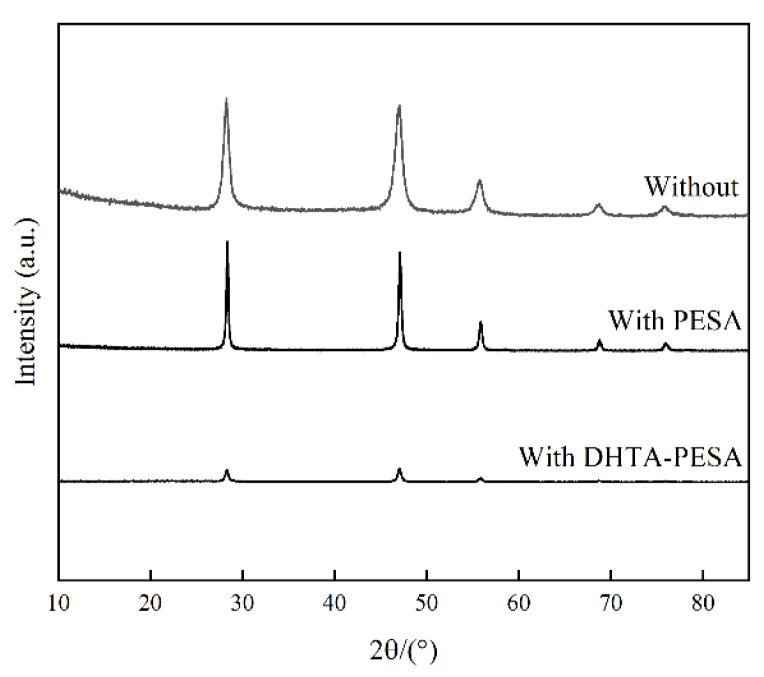
XRD spectra of CaF_2_ scale samples without the addition of scale inhibitors and with the addition of PESA and DHTA-PESA.

The interaction between the polymer and CaF_2_ can be reflected by the energy barrier through X-ray diffraction (XRD) analysis of CaF_2_ crystals. Scale samples with and without the addition of 100 mg/L scale inhibitor were collected and scanned using an XRD scanner at a scanning rate of 15°/min in the range of 5–85°. The results are shown in Figure 17.

When no scale inhibitor was added, typical CaF_2_ diffraction peaks were observed at 28.23°, 46.96°, 55.72°, 68.86°, and 75.84° in the CaF_2_ scale sample. The addition of DHTA-PESA did not change the positions of these diffraction peaks compared to the blank, indicating that DHTA-PESA influences the formation process of CaF_2_ crystals without altering their final crystalline form [37]. In terms of peak characteristics, higher and sharper peaks indicate better crystallinity and more complete crystal formation, which in this experiment promotes CaF_2_ precipitation. The introduction of scale inhibitors decreases the peak intensity, disrupts crystal ordering, and results in loose, porous structures or hinders complete crystal formation.

DHTA-PESA has a more significant impact on the growth of CaF_2_ crystals and demonstrates stronger targeting ability against CaF_2_ scale. The introduction of more carboxyl and hydroxyl groups in DHTA-PESA enables it to bind more Ca^2+^ and form soluble complexes. These functional groups also induce distortion in CaF_2_ crystal formation, leading to the appearance of unstable crystal phases. Such unstable crystal structures are easily washed away by water flow, thereby achieving better scale inhibition effects [14,38].

Through static and dynamic experiments, as well as SEM and XRD analysis of scale samples, it was found that the inhibitory effect of DHTA-PESA as a scale inhibitor on CaF_2_ scale is primarily attributed to the introduction of carboxyl and hydroxyl groups. These groups exhibit strong chelating and dispersing effects with Ca^2+^. Meanwhile, during the nucleation process, DHTA-PESA can occupy active sites on the crystal surface, causing distortion in CaF_2_ crystal formation. The addition of carboxyl and hydroxyl groups enhances the three major effects of the scale inhibitor, thereby demonstrating a stronger inhibitory effect on CaF_2_ scale.

#### 3.4.3. XPS Analysis

**Figure 18:**
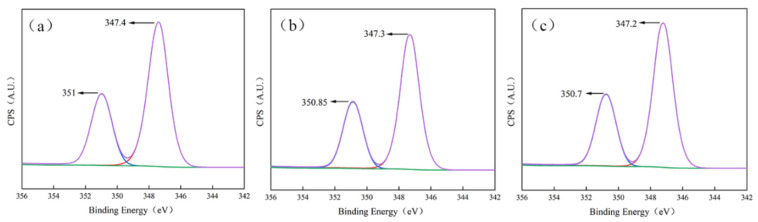
XPS image of CaF_2_ scale (concentration 100 mg/L) (**a**) without adding scale inhibitor, (**b**) CaF_2_ scale after adding PESA, and (**c**) CaF_2_ scale after adding DHTA-PESA.

To further investigate the adsorption characteristics of DHTA-PESA, X-ray photoelectron spectroscopy (XPS) analysis was conducted on the CaF_2_ scale formed in the static experiment. The results are shown in Figure 18.

When no scale inhibitor is added, significant characteristic peaks of CaF_2_ scale appear at 351 eV and 347.4 eV, respectively. After adding PESA, the peak positions shift to 350.85 eV and 347.3 eV due to changes in the chemical environment around Ca^2+^. Further addition of DHTA-PESA causes the peak positions to shift to 350.7 eV and 347.2 eV, respectively. This suggests that the introduced carboxyl groups enhance chelation with Ca^2+^, alter the electron density around Ca^2+^, and promote the migration of calcium peak positions [39].

Compared with PESA, DHTA-PESA has a more pronounced effect on the peak position of calcium, indicating stronger chelating ability with Ca^2+^ and greater inhibition of CaF_2_ scale. The XPS data, combined with SEM and XRD results, show that DHTA-PESA can adsorb on the surface of CaF_2_ scale, influencing crystal surface morphology and growth rate. This effectively inhibits the formation, growth, and deposition of crystals in aqueous solution.

## 4. Conclusions

The synthesized DHTA-PESA is a phosphorus-free, nitrogen-free, green, and environmentally friendly scale inhibitor. It not only exhibits excellent inhibitory efficiency against calcium fluoride (CaF_2_) scale but also aligns with the concept of green and sustainable development. Static experimental results demonstrated that DHTA-PESA achieves the optimal scale inhibition rate under the following conditions: concentration of 100 mg/L, temperature of 50 °C, Ca^2+^ concentration of 240 mg/L, heating duration of 6 h, and pH value of 7. Dynamic experiments indicated that DHTA-PESA possesses a superior dispersion capacity, which can effectively alleviate membrane pore blockage. Mechanistic analysis revealed that the scale-inhibiting effect of DHTA-PESA on CaF_2_ scale stems from its dual functions of chelation and dispersion: on one hand, it can chelate Ca^2+^ ions in water; on the other hand, it enables CaF_2_ crystals to carry the same charge, thereby inducing electrostatic repulsion between the crystals to prevent aggregation. Meanwhile, DHTA-PESA can also cause distortion of CaF_2_ crystals, ultimately ensuring that microcrystals remain stably dispersed in water and hardly form scale deposits.

## Figures and Tables

**Table 1:** Viscosity data for PESA.

Number of Times	t_0_	t_1_	η_r_	η_sp_	[η]	M_η_
1	83.34	85.61	1.0272	0.0272	0.0269	497.71
2	83.42	85.63	1.0264	0.0265	0.0263	471.07
3	83.38	85.58	1.0263	0.0264	0.0262	467.30

**Table 2:** Viscosity data for DHTA-PESA.

Number of Times	t_0_	t_1_	η_r_	η_sp_	[η]	M_η_
1	83.34	87.35	1.0481	0.0481	0.0474	1532.33
2	83.42	87.45	1.0483	0.0483	0.0476	1544.49
3	83.38	87.44	1.0487	0.0487	0.0479	1568.69

**Table 3:** Central water quality table.

Item	Ca^2+^ (mg/L)	Cl^−^ (mg/L)	K^+^ (mg/L)	F^−^ (mg/L)	Mg^2+^ (mg/L)	Na^+^ (mg/L)	pH
Actual reclaimed water	250.96	907.5	45.89	14.0	73.11	745.69	7.45~8.03

## Data Availability

The original contributions presented in this study are included in the article. Further inquiries can be directed to the corresponding authors.
